# Role of apolipoprotein O in autophagy via the p38 mitogen-activated protein kinase signaling pathway in myocardial infarction

**DOI:** 10.1016/j.clinsp.2022.100046

**Published:** 2022-05-16

**Authors:** Yue Liu, Zhiping Xiong, Wei Zhou, Yuxin Chen, Qing Huang, Yanqing Wu

**Affiliations:** Nanchang University Second Affiliated Hospital, Cardiovascular Medicine, Nanchang City, Jiangxi Province, PR China

**Keywords:** Apolipoprotein O, Autophagy, Apoptosis, Myocardial Infarction, p38MAPK

## Abstract

**Objective:**

To explore the role and possible mechanisms of action of apolipoprotein O (APOO) in autophagy in Myocardial Infarction (MI) *in vivo* and *in vitro*.

**Methods:**

Differential gene expression and single Gene Set Enrichment Analysis (GSEA) were used to evaluate MI-related candidate genes. Animal and cell MI models were established. Sh-APOO, si-APOO, and SB203580 were used to inhibit the expression of APOO or p38MAPK. Western blot and qRT-PCR were used to analyze the expression levels of the target protein or mRNA. Apoptosis was observed using the TUNEL assay. The plasma concentrations of CK-MB and cTn-I in humans and mice were determined.

**Results:**

In the GSE23294 dataset, APOO mRNA was highly expressed in the left ventricle of mice with MI; GSEA revealed that APOO was positively correlated with p38MAPK, autophagy, and apoptosis. The plasma concentration of APOO in patients with MI was significantly higher than that in healthy subjects. The expression of APOO, Beclin-1, LC3, and Bax in mouse and AC16 cell MI models increased, while the level of Bcl-2 decreased. After silencing the APOO gene, the expression of APOO was downregulated; meanwhile, changes in autophagy, apoptosis and myocardial cell injury were reversed *in vivo* and *in vitro*. Furthermore, autophagy was alleviated after AC16 cells were treated with SB203580.

**Conclusions:**

The increased APOO expression in mouse and cell MI models may activate autophagy and apoptosis by regulating the p38MAPK signaling pathway, thus aggravating the myocardial injury.

## Highlights

• APOO was highly expressed in the left ventricle of mice with myocardial infarction.
• Increasing of APOO may activate autophagy and apoptosis in myocardial infarction.
• The regulation of APOO in autophagy and apoptosis was regulated by p38MAPK signaling pathway.

## Introduction

Myocardial Infarction (MI) is a common pathological condition that affects human health and is usually caused by the narrowing of the coronary artery due to atherosclerosis. The resulting persistent ischemia leads to severe necrosis of the cardiomyocytes.^1^ Hypertension, diabetes, and dyslipidemia can also lead to MI.^2^ Studies have shown that the accumulation of reactive oxygen species, upregulated expression of adhesion molecules, and increased blood coagulation may lead to dyslipidemia and subsequently to MI.^3^ Abnormal regulation of several signaling pathways can be found during MI, including Transforming Growth Factor-β (TGF-β), p38 Mitogen-Activated Protein Kinase (p38MAPK), and Phosphoinositide-3-Kinase (PI3K)/Akt.^4^^,^^5^ Of these signaling pathways, p38MAPK can regulate inflammation, oxidative stress, apoptosis, and autophagy.^6^ p38MAPK plays an important role in phosphorylating nuclear transcription factors and regulating gene expression. It has also been reported that the inhibition of p38MAPK can prevent MI.^7^

Apolipoprotein O (APOO) was discovered in 2006; it is expressed in many human tissues. In addition to acting as a secretory protein outside the cell, APOO can also be retained in the cell and forms a constituent of the intracellular membranes, including lipid droplets and mitochondrial membranes.^8^ Yu et al. found that the average plasma APOO concentration in healthy subjects was 2.21±0.83 µg/mL, while that in Coronary Artery Disease (CAD) patients was 4.94±1.59 µg/mL.^9^ There was no correlation between secretory APOO and high-density lipoprotein cholesterol levels or other lipid parameters, suggesting that APOO mainly functions in cells.^9^ Several studies have shown that APOO is overexpressed in the hearts of diabetes patients^8^ and in the hearts of animals fed a high-fat diet.^10^

APOO may play different roles in the mitochondrial inner membrane system.^11^^,^^12^ APOO is considered to be a regulatory mediator in the protective mechanism preventing cardiac adipocyte apoptosis,^13^ but the overexpression of APOO promotes apoptosis, and the mRNA levels of APOO are positively correlated with the expression of the pro-apoptotic factor Bax in the human heart.^11^ Pathological overexpression of APOO may induce mitochondrial metabolic disorders and drive cells into a vicious cycle, resulting in mitochondrial changes, apoptosis, and cell death. The resulting increase in mitochondrial synthesis may be an adaptive response that balances the changes or degradation of mitochondria in antiphagocytic vacuoles and multilamellar bodies and attempts to restore decreased cellular energy.^11^ One study revealed that increased expression of APOO in cultured cardiomyocytes, from both transgenic mice and humans, is associated with excessive activation of lipid metabolism, and this hyperactive lipid metabolism is related to the activation of programmed cell death and autophagy.^14^ However, the regulatory effects and mechanism of APOO on autophagy in MI are not clear.

In this study, the authors hypothesized that APOO plays an important role in MI and that the underlying mechanism is mediated through the p38MAPK signaling pathway, thus aggravating myocardial injury via autophagy and apoptosis. The role of APOO in MI was studied by establishing mouse and cellular MI-related models and silencing the expression of APOO. The authors found that APOO mediates autophagy through the P38MAPK signaling pathway in MI, which aggravates MI.

## Materials and methods

### Antibodies and reagents

Human CK-MB ELISA kit (D711191), Mouse CK-MB ELISA kit (D721065), human cTn-I ELISA kit (D711127), and mouse cTn-I ELISA kit (D721149) were purchased from Sangon Biotech. The human APOO ELISA kit was purchased from Shanghai SHUANGYING Biological Technology Co. The lactate dehydrogenase assay kit (LDH, A020-2) was purchased from Nanjing Jiancheng Bioengineering Institute. The Cell Counting kit-8 assay (CCK-8, C6005) was obtained from US Everbright Inc. SB203580, a P38MAPK inhibitor was purchased from Invitrogen (Carlsbad, CA, USA). Antibodies against LC3 (#12741), Beclin-1 (#3495), Bax (#2772), Bcl-2 (#3498), phosphorylated (p)-P38MAPK (#4511), p38MAPK (#8690), and β-actin (#3700) were purchased from Cell Signaling Technology. Antibodies against APOO (A09879) were purchased from Boster Biological Technology Co. The procedures were in accordance with the Helsinki Declaration.

### Differential gene expression

The gene expression profiles from the GSE23294 array and related clinical data can be downloaded from the Gene Expression Omnibus (GEO, https://www.ncbi.nlm.nih.gov/geo/). This dataset includes gene expression profiles from 10 MI model mice and 10 non-MI model mice. Differentially expressed proteins were screened according to the criteria of | logFC | > 1 and adj.*p* < 0.001.

### Gene set enrichment analysis (GSEA)

GSEA (version 4.0.3; Broad Institute, USA) was used to analyze the genes that were differentially expressed between the high- and low-risk groups. A total of 1,000 permutations were selected, and Affymetrix was used as the chip platform for the calculation of the Normalized Enrichment Score (NES). A normal p-value < 0.05 and false discovery rate (FDR q-value) < 0.25 were considered indicators of significant enrichment.

### Establishment of the mouse model of MI

Male C57BL/6 mice (body mass 21‒25g, 8-weeks old) were purchased from SiPeiFu Biotechnology Co., Ltd. (Beijing, China). The mice were fed in a pathogen-free room with a temperature of 25‒28°C, relative humidity of 40%‒60%, and a light-dark cycle of 10:14 hours. After 1-week of acclimation, acute MI was induced by ligating the anterior descending branch of the coronary artery. Mice were anesthetized by intraperitoneal injection of pentobarbital sodium (50 mg/kg) and ventilated with positive pressure on an animal ventilator to help the mice breathe. Thoracotomy was performed through the third or fourth intercostal space on the left side of the mouse, and the pericardium was cut open to expose the heart. At the junction of the left atrium and left ventricle, the anterior descending branch of the left coronary artery was ligated with a 7‒0 prolene suture to induce MI. If the tissue downstream of the coronary artery ligation turned white, the heartbeat of the mouse weakened, and the ST-T segment was elevated excessively in the electrocardiogram, then MI was considered to be successfully established. The ribs, muscles, and skin were then sutured, and the trachea was extubated after spontaneous breathing. The sham operation control group only underwent thoracotomy, and the coronary artery was not ligated. After surgery, mice were placed in a 30°C incubator for at least 30 min before moving back to the cage. The infection and activity of the mice were monitored daily. Four weeks after the MI, the mice were anesthetized and examined using echocardiography. The mice were sacrificed, and blood samples and heart tissue were collected. One part of the heart was fixed with formaldehyde, and the remaining parts were snap-frozen in liquid nitrogen and stored at -80°C for real-time Polymerase Chain Reaction (PCR) and western blot analysis. All experimental procedures were performed in compliance with the guidelines for the Care and Use of Laboratory Animals (NIH Publication n° 85‒23, revised 1996), and the experimental design was approved by the Ethics Committee of The Second Affiliated Hospital of Nanchang University.

### APOO knockdown by lentivirus injection

APOO knockdown lentivirus (sh-APOO: 5’- GGTTAGACAGCTATGACTA-3’) and control virus were designed and prepared by GenePharma Corporation (Shanghai, China). The lentivirus vectors were stored at -80°C. GC APOO shRNA (1 × 10^7^ GC APOO shRNA) was injected into six sites in the left ventricular anterior wall 2-weeks before ligation of the left anterior descending coronary artery, and the control shRNA mice received the same volume of control shRNA, as previously described.^15^

### Cell culture and treatment

The human cardiomyocyte line AC16 was obtained from ATCC (Manassas, VA, USA). The AC16 cells were maintained in Dulbecco's modified Eagle's medium (DMEM, Gibco, NY, USA) with 10% Fetal Bovine Serum (FBS), 100 U/mL penicillin, and 100 μg/mL streptomycin (all from Invitrogen, Carlsbad, CA, USA) at 37°C in a humidified incubator containing 5% CO_2_ and 95% air. The medium was changed daily. The cells were starved in a serum-free medium for 12h prior to experimentation. To imitate myocardial ischemia, the AC16 cells were exposed to hypoxic conditions with 94% N_2_, 5% CO_2_, and 1% O_2_ for 12h, 24h, and 36h at 37°C.

### Cell transfection

Small interfering RNAs targeting APOO (si-APOO: 5‘-UGUCUAACCCCCAUUGAACCA-3’) and a Scrambled Negative Control (si-NC) were designed and synthesized by RIBOBIO Co., Ltd. (Guangzhou, China). After conventional culture for 24h in six-well plates, transient transfection of AC16 cells with the oligonucleotides was carried out using Lipofectamine 3000 reagent (Invitrogen, Carlsbad, CA, USA), followed by hypoxia treatment for 24h.

### Enzyme-linked immunosorbent assay (ELISA)

The whole blood of patients and mice was collected using an anticoagulant tube within 24h after admission and after anesthesia, respectively. Within 30 min, whole blood samples were centrifuged at 3,500 rpm at 4°C for 15 min, and then the plasma was collected. The plasma levels of CK-MB, cTnI, and APOO were detected using the corresponding ELISA kits according to the manufacturer's instructions.

### Measurement of cell viability

Cell viability was determined using a CCK-8 assay kit. The AC16 cells were suspended in DMEM and cultured in 96-well plates at a density of 1 × 10^4^ cells per well. After treatment, the AC16 cells were washed, then non-FBS DMEM (100 μL/well) and CCK-8 solution (10 μL/well) was added, and the cells were incubated at 37°C for an additional 2h. The absorbance of each well was measured at 450 nm using a microplate reader (Bio-Rad 680, Hercules, CA, USA). Cell viability (%) = (A_treatment_ − A_blank_)/(A_control_ − A_blank_)  ×  100.

### Cellular injury assay

The cellular injury was evaluated using an LDH assay kit, according to the manufacturer's protocol. Briefly, 0.2% Triton X-100 was used to lyse the cells. After centrifugation, the supernatants were harvested and treated for 30 min with 100 μL of LDH reaction solution. The absorbance of each well was measured at 450 nm using a microplate reader (Bio-Rad 680, Hercules, CA, USA). Cytotoxicity (%) = (A_treatment_ − A_control_)/(A_max_ − A_control_)  ×  100.

### Cell apoptosis detection

The AC16 cells were seeded on glass coverslips. After treatment, the slides were fixed in 4% paraformaldehyde and permeabilized with 0.2% Triton X-100. Apoptotic cells were detected using a Terminal Deoxyribonucleotidyl Transferase (TdT)-mediated biotin-16-dUTP Nick End Labeling (TUNEL) kit. The TUNEL assay was performed according to the manufacturer's instructions. Images were captured using fluorescence microscopy at 400 ×  magnification.

### Western blot analysis

The mouse myocardial tissue and AC16 cardiomyocytes were lysed with RIPA lysis solution (Beyotime, Shanghai, China) at 4°C. After determining the protein concentration using the BCA kit, the protein extract (20‒40 μg) was added to 12% sodium dodecyl sulfate-polyacrylamide gel electrophoresis and transferred to a polyvinylidene fluoride membrane. The membrane was incubated with primary antibodies (APOO 1:1000, Beclin-1 1:3000, LC3 1:3000, Bax 1:3000, Bcl-2 1:2000, and p38MAPK 1:2000) overnight at 4°C. The next day, at room temperature, the membrane was incubated with horseradish peroxidase-coupled secondary antibody (anti-rabbit IgG 1:5000, anti-mouse IgG: 1:5000) for 2h. The intensities of the protein bands were quantified using the ChemiDocTM Touch Imaging System and normalized to β-actin levels.

### Quantitative real-time PCR (qRT-PCR)

The CFX96 real-time PCR detection system (Bio-Rad) was used to quantify gene expression by SYBR Green real-time PCR. First, total RNA was extracted using TRIzol reagent. The PrimeScript RT-PCR kit (Takara Biotechnology, Dalian, China) was used for qRT-PCR analysis of mRNA. The primers used are listed in Table 1. The results were standardized to the control value for β-actin.

**Table 1:** Primer sequences of related genes.

		Forward Primer (5′-3′)	Reverse Primer (5′-3′)
Human	APOO	CCTTCAAAGTCTATGCAGCACC	TTCGATTGACCCTCAGGAACT
	LC3B	AAGGCGCTTACAGCTCAATG	CTGGGAGGCATAGACCATGT
	Beclin1	ACCTCAGCCGAAGACTGAAG	AACAGCGTTTGTAGTTCTGACA
	BAX	CCCGAGAGGTCTTTTTCCGAG	CCAGCCCATGATGGTTCTGAT
	BCL2	GGTGGGGTCATGTGTGTGG	CGGTTCAGGTACTCAGTCATCC
	β-actin	CATGTACGTTGCTATCCAGGC	CTCCTTAATGTCACGCACGAT
	Apoo	AACATCTCACAACTCCGACATC	TGTCTACTCCCCACTGGACAA
	Lc3b	CGCTTGCAGCTCAATGCTAAC	CTCGTACACTTCGGAGATGGG
Mouse	beclin1	TCAGCCGGAGACTCAAGGT	CACAGCGGGTGATCCACATC
	Bax	CCGGCGAATTGGAGATGAACT	CCAGCCCATGATGGTTCTGAT
	Bcl2	GAGAGCGTCAACAGGGAGATG	CCAGCCTCCGTTATCCTGGA
	β-actin	GTGACGTTGACATCCGTAAAGA	GCCGGACTCATCGTACTCC

### Statistical analysis

Data are expressed as mean ± standard deviation. A student's *t*-test was performed to compare the differences between two groups, and a one-way analysis of variance was used for multiple groups. Differences were considered statistically significant at *p* < 0.05. Statistical analysis was performed using GraphPad Prism 7.0 software (GraphPad Software, San Diego, USA).

## Results

### The levels of APOO, autophagy, and apoptosis were increased after MI

**Fig. 1:**
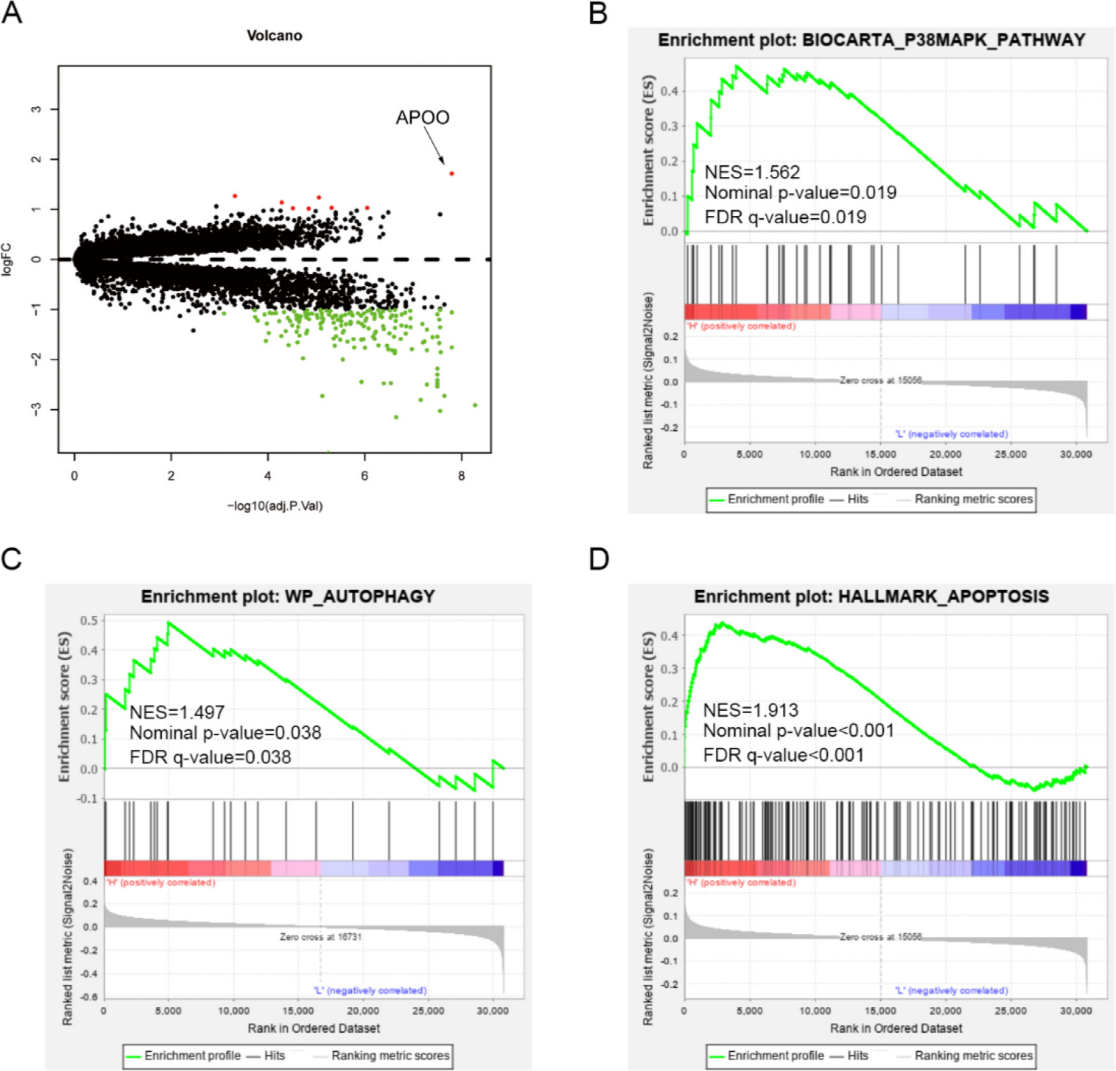
The volcano plot is used to show the differentially expressed genes between mice of MI model group and non-MI model (A). Gene Set Enrichment Analysis (GSEA) was used to analyse the signaling pathways enrichment in different groups (B‒D).

Studies have shown that the plasma concentration of APOO in patients with CAD is higher than that in healthy subjects,^9^ suggesting that the APOO gene may be associated with CAD and that MI is a severe CAD. By analyzing the differentially expressed genes between the MI and non-MI groups in the GSE23294 dataset, APOO mRNA was found to be highly expressed in the left ventricle of MI model mice (Fig. 1A). To analyze the biological functions of APOO and the signaling pathways regulated by APOO, GSEA was used; results revealed that APOO expression was positively correlated with p38MAPK, autophagy, and apoptosis (Fig. 1B-D).

**Fig. 2:**
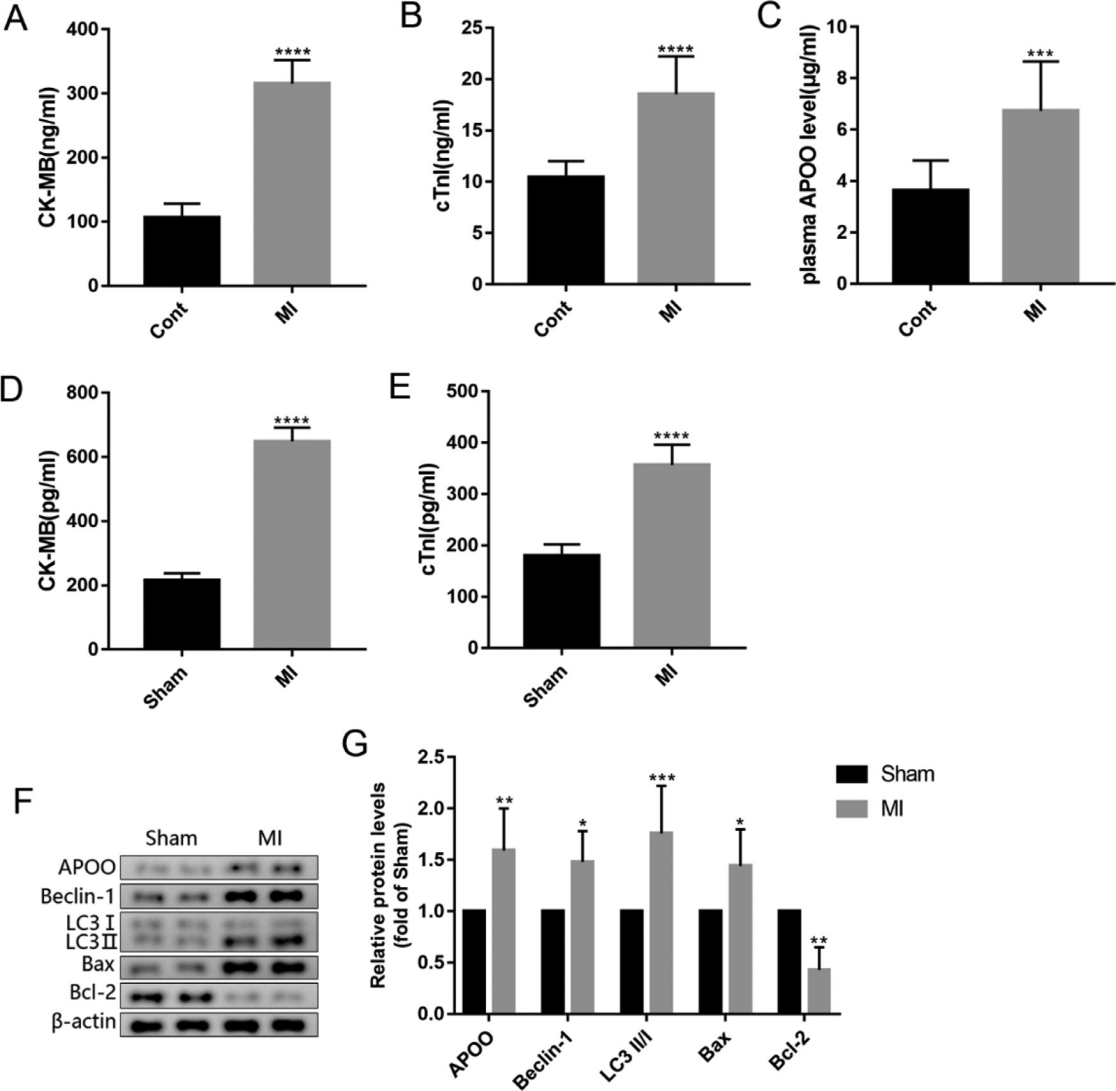
The plasma level of CK-MB (A), cTnI (B) and APOO (C) in patients with miocardial infarction (MI) and healthy subjects. The plasma level of CK-MB (D) and cTnI (E) in mice of different groups. Western blot assay of APOO, Beclin1, LC3, Bax, and Bcl-2 expression in mice of sham and MI group (F). Quantification of target protein in different mice (G). All results are expressed as the mean ± SD, *n* = 3. *p < 0.05, ^⁎⁎^*p* < 0.002, ^⁎⁎⁎^*p* < 0.0002, ^⁎⁎⁎⁎^*p* < 0.0001 versus cont (patients) or Sham (mice) group.

To further clarify the relationship between APOO and MI, the authors analyzed the plasma APOO concentration after MI. ELISA results showed that the plasma concentrations of CK-MB, cTnI, and APOO in patients with MI were higher than those in healthy controls (Fig. 2A-C). In the MI mouse model, the plasma concentrations of CK-MB and cTnI in the MI group were higher than those in the sham group (Fig. 2D, E). Western blot analysis showed that the protein expression level of APOO in the myocardial tissue was also higher than that in the sham group (Fig. 2F, G). In addition, the protein expression levels of Beclin-1, LC3, and Bax in the MI group were higher than those in the sham group, while the levels of Bcl-2 protein were lower in the MI group than in the sham group (Fig. 2F, G), indicating an increase in myocardial autophagy and apoptosis in MI mice.

**Fig. 3:**
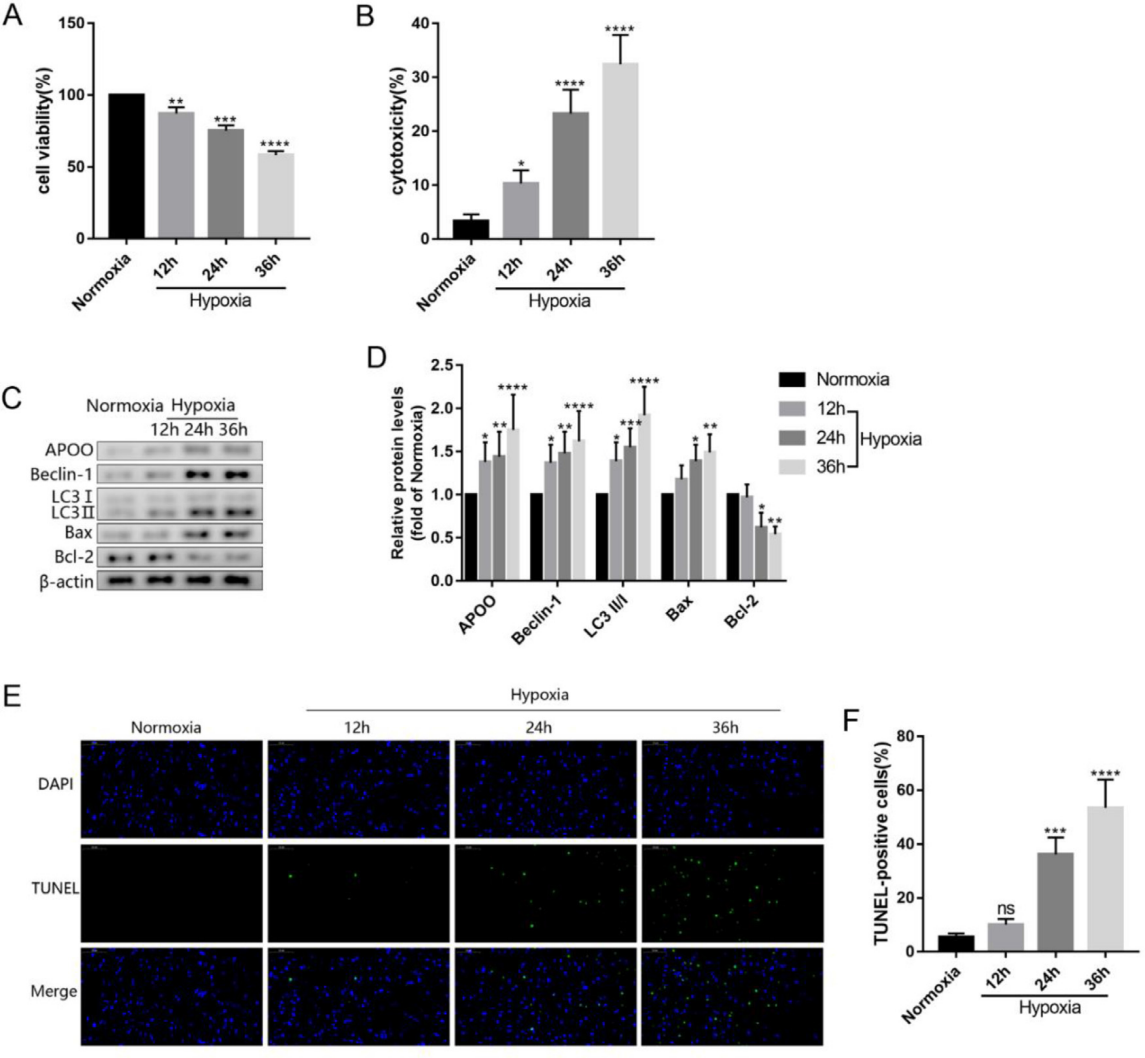
The cell viability (A) and cytotoxicity (B) of AC16 cell in different group. Western blot assay of APOO, Beclin1, LC3, Bax, and Bcl-2 expression in AC16 cell (C). Quantification of target protein in AC16 cell after different treatment (D). Immunofluorescence assay of TUNEL (E) and TUNEL positive cells counting in different group (F), the scale bar represents 50 µm. All results are expressed as the mean ± SD, *n =* 3. *p < 0.05, ^⁎⁎^*p* < 0.002, ^⁎⁎⁎^*p* < 0.0002, ^⁎⁎⁎⁎^*p* < 0.0001 versus Normoxia group.

To further verify these findings, the authors subjected the AC16 cells to hypoxia for 12h, 24h, and 36h. Cell activity decreased, and cytotoxicity increased in a time-dependent manner after hypoxia treatment (Fig. 3A, B). Furthermore, the protein levels of APOO, Beclin-1, LC3, and Bax in the AC16 cells increased at all time points after hypoxia treatment, while the protein levels of Bcl-2 decreased (Fig. 3C, D). Results of the TUNEL assay also showed that apoptosis increased after hypoxia (Fig. 3E, F). These findings indicate that the concentration of APOO in myocardial tissue was increased, autophagy was activated, and apoptosis was increased in the mouse model of MI.

### Inhibition of autophagy by APOO gene silencing in mice

**Fig. 4:**
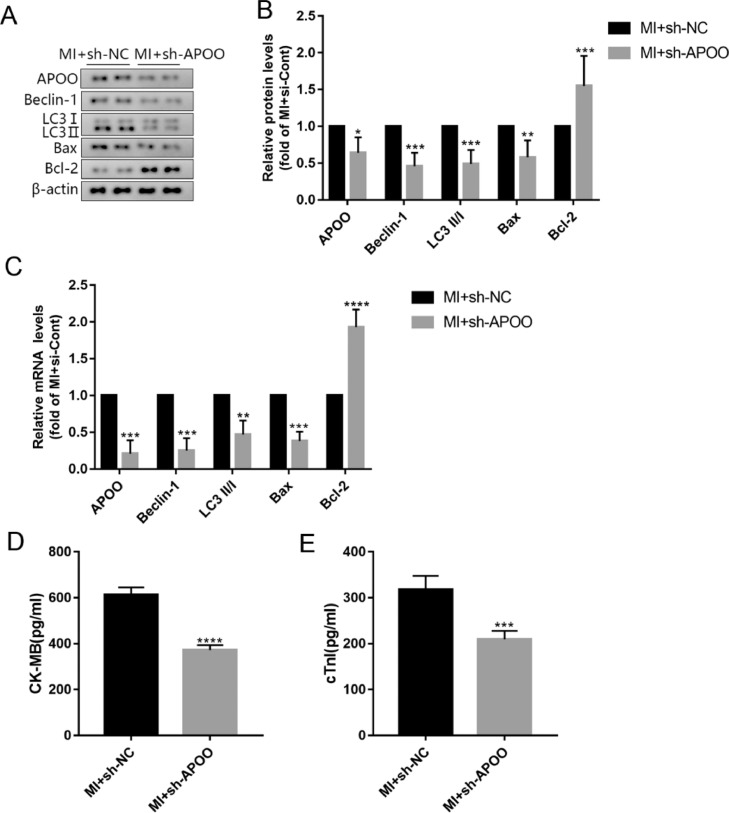
Western blot assay of APOO, Beclin1, LC3, Bax, and Bcl-2 expression of mice in MI+sh-NC group and MI+sh-APOO group (A). Quantification of target protein in different group (B). qRT-PCR assay of APOO, Beclin1, LC3, Bax, and Bcl-2 expression of mice in MI+sh-NC group and MI+sh-APOO group (C). The level of CK-MB (D) and cTnI (E) in mice of different groups. All results are expressed as the mean ± SD, *n =* 3. *p < 0.05, ^⁎⁎^*p* < 0.002, ^⁎⁎⁎^*p* < 0.0002, ^⁎⁎⁎⁎^*p* < 0.0001 versus MI+sh-NC group.

The above results have shown that autophagy and APOO expression were increased after MI. APOO may also regulate autophagy.^11^^,^^14^ To study the potential effects of APOO on autophagy in MI, the authors treated mice with sh-APOO lentivirus and analyzed the expression levels of autophagy-related proteins in the myocardial tissue. The levels of APOO protein and mRNA in the myocardial tissue of MI+sh-APOO mice were significantly lower than those in MI+sh-NC mice (Fig. 4A-C). The protein and mRNA levels of autophagy markers Beclin-1 and LC3 were significantly decreased (Fig. 4A-C). The expression of the apoptosis-related marker Bax was significantly decreased, while the expression of Bcl-2 was significantly increased (Fig. 4A-C). At the same time, the plasma concentrations of CK-MB and cTnI in the MI+sh-APOO group were lower than those in the MI+sh-NC group (Fig. 4D, E). These findings suggest that the expression of APOO may be related to cardiomyocyte autophagy and apoptosis in MI.

### Inhibition of autophagy by APOO gene silencing in cells

**Fig. 5:**
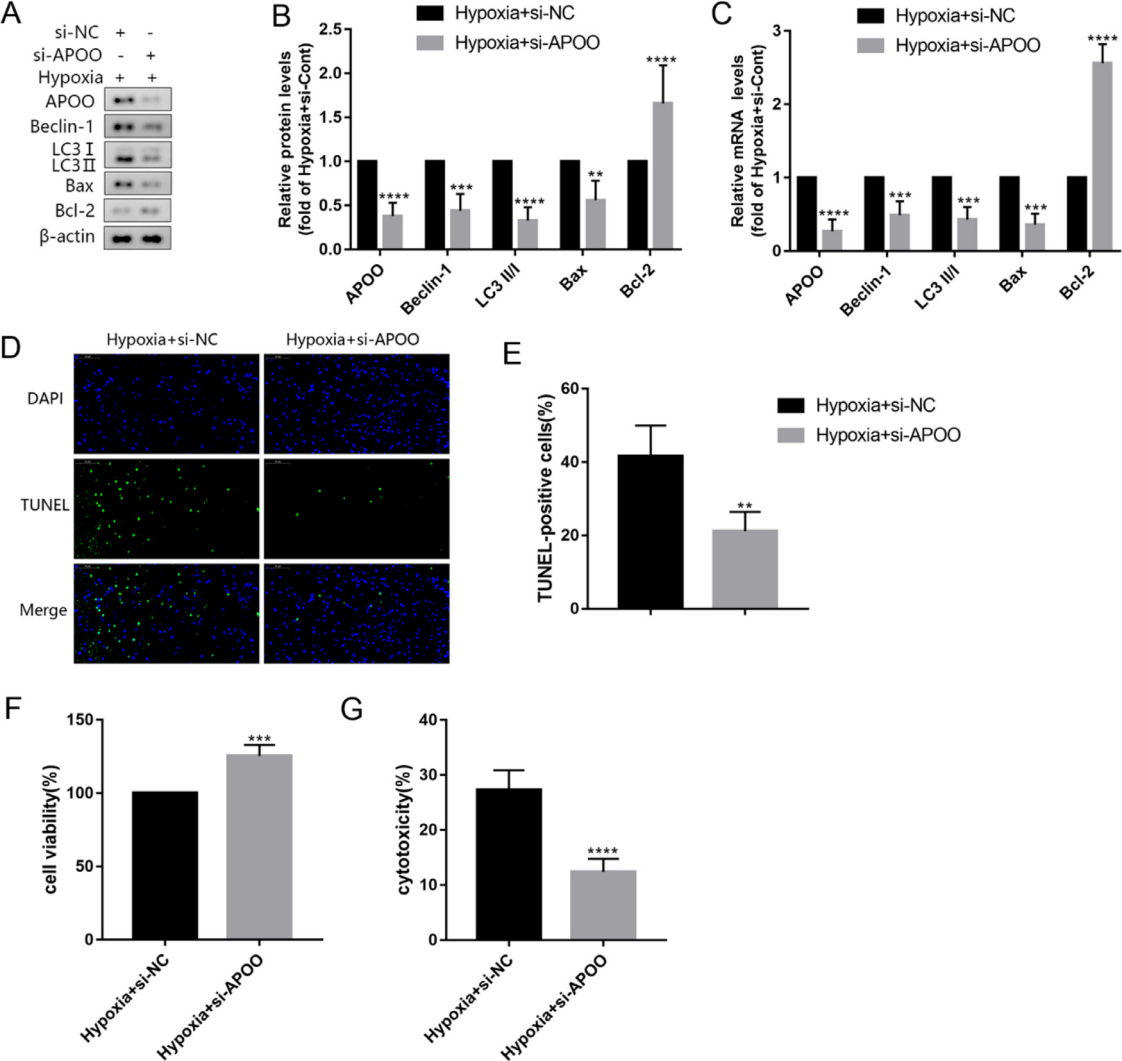
Western blot assay of target protein level of AC16 cells in Hypoxia+si-NC group and Hypoxia+si-APOO group (A). Quantification of target protein in different group (B). qRT-PCR assay of target gene expression of AC16 cells in Hypoxia+si-NC group and Hypoxia+si-APOO group (C). Immunofluorescence assay of TUNEL (D) and TUNEL positive cells counting in different group (E), the scale bar represents 50 µm. The cell viability (F) and cytotoxicity (G) of AC16 cell in different group. All results are expressed as the mean ± SD, *n =* 3. ^⁎⁎^*p* < 0.002, ^⁎⁎⁎^*p* < 0.0002, ^⁎⁎⁎⁎^*p* < 0.0001 versus Hypoxia+si-NC group.

To further determine the relationship between APOO, autophagy, and apoptosis, the authors transfected AC16 cells with si-APOO and silenced the expression of APOO in AC16 cells. Similarly, the authors found that the levels of APOO protein and mRNA in AC16 cells in the hypoxia+si-APOO group were lower than those in the hypoxia+si-NC group (Fig. 5A-C), and the protein and mRNA levels of Beclin-1 and LC3 were significantly decreased in the hypoxia+si-APOO group (Fig. 5A-C). The expression levels of Bax decreased significantly, whereas the expression levels of Bcl-2 increased significantly (Fig. 5A-C). Results of the TUNEL assay also showed that apoptosis was decreased in the hypoxia+si-APOO group (Fig. 5D, E). In addition, the cell viability increased, and cytotoxicity decreased in the hypoxia+si-APOO group (Fig. 5F). These results suggest that the increased expression of APOO in MI may lead to apoptosis by activating autophagy, thus aggravating cardiomyocyte injury and death.

### Activation of the p38MAPK signaling pathway by APOO is essential for MI

**Fig. 6:**
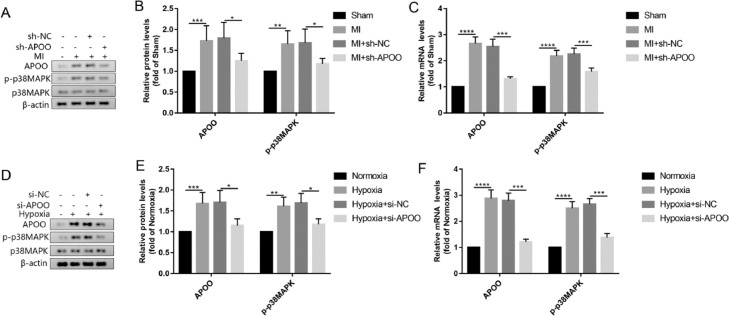
Western blot assay of APOO and p-p38MAPK expression level in mice of Sham, MI, MI+sh-NC, and MI+sh-APOO group (A). Quantification of target protein in different group (B). qRT-PCR assay of APOO and p-p38MAPK expression in mice of different group (C). Western blot assay of APOO and p-p38MAPK expression level in AC16 cells of Normoxia, Hypoxia, Hypoxia+si-NC, and Hypoxia+si-APOO group (D). Quantification of target protein in different group (E). qRT-PCR assay of APOO and p-p38MAPK expression in AC16 cells of different group (F). All results are expressed as the mean ± SD, *n =* 3. ^⁎⁎^*p* < 0.002, ^⁎⁎⁎^*p* < 0.0002, ^⁎⁎⁎⁎^*p* < 0.0001 versus Sham (mice) or Normoxia (AC16 cell) group.

To explore the mechanism by which APOO regulates autophagy, the authors measured the expression level of p38MAPK in animals and cells. Western blot assay showed that the levels of p38MAPK phosphorylation and APOO protein expression in myocardial tissue of the MI group were significantly higher than those in the sham group (Fig. 6A, B). However, after the sh-APOO treatment of MI mice, the expression levels of p-p38MAPK and APOO in the myocardium decreased (Fig. 6A, B). To further verify these findings, qRT-PCR was used to detect the mRNA expression levels of p-p38MAPK and APOO in mouse myocardium, and the results were consistent with those obtained by western blotting (Fig. 6C). In cell experiments, the protein and mRNA expression levels of p-p38MAPK and APOO in the hypoxia group were higher than those in the normoxia group (Fig. 6D-F). However, the expression of APOO and p-p38MAPK decreased significantly after si-APOO interference (Fig. 6D-F). In conclusion, these results suggest that APOO silencing may inhibit the phosphorylation of p38MAPK.

### The p38MAPK signaling pathway participates in autophagy activation in MI

**Fig. 7:**
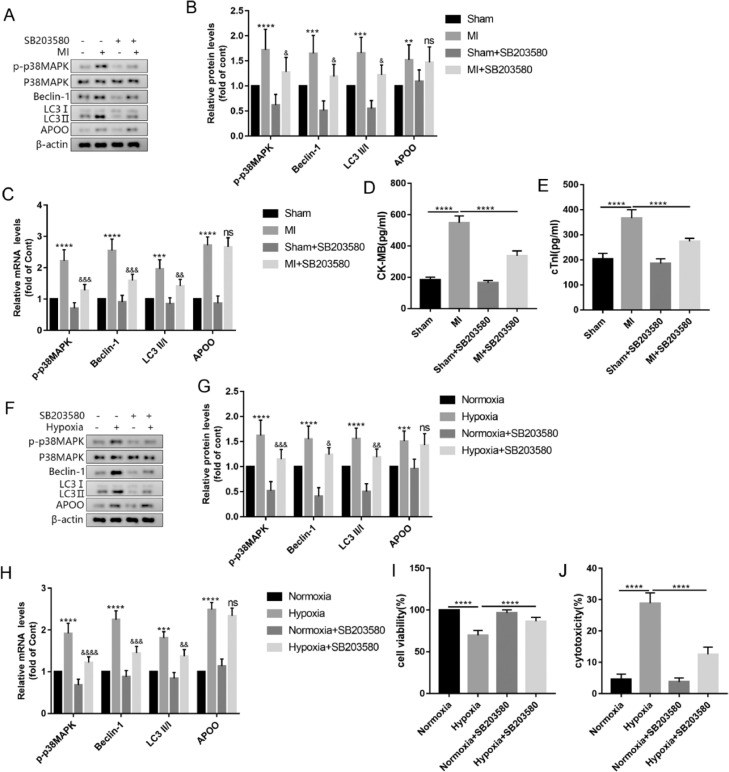
Western blot assay of p-p38MAPK, Beclin1, LC3, and APOO expression of mice in Sham, MI, Sham+SB203580 and MI+SB203580 group (A). Quantification of target protein in different group (B). qRT-PCR assay of p-p38MAPK, Beclin1, LC3, and APOO expression of mice in different group (C). The level of CK-MB (D) and cTnI (E) in mice of different groups. Western blot assay of p-p38MAPK, Beclin1, LC3, and APOO level of AC16 cells in Normoxia, Hypoxia, Normoxia+SB203580, and Hypoxia+SB203580 group (F). Quantification of target protein in different group (G). qRT-PCR assay of p-p38MAPK, Beclin1, LC3, and APOO expression of AC16 cells in different group (H). The cell viability (I) and cytotoxicity (J) of AC16 cells in different group. All results are expressed as the mean ± SD, *n =* 3. ^⁎⁎^*p* < 0.002, ^⁎⁎⁎^*p* < 0.0002, ^⁎⁎⁎⁎^*p* < 0.0001 versus Sham (mice) or Normoxia (AC16 cell) group. ^&^*p* < 0.05, ^&&^*p* < 0.002, ^&&&^*p* < 0.0002, ^&&&&^*p* < 0.0001 versus MI (mice) or Hypoxia (AC16 cell) group.

To explore whether the p38MAPK signaling pathway is a necessary step in APOO-mediated autophagy, the authors used SB203580, a common p38MAPK inhibitor, to treat mice and AC16 cells. The results showed that compared with the MI group, the protein and mRNA levels of p-p38MAPK in the myocardial tissue of the MI+SB203580 group were significantly decreased and the expression levels of autophagy markers Beclin-1 and LC3 were decreased, but the expression level of APOO showed no significant change (Fig. 7A-C). In addition, the plasma levels of CK-MB and cTnI in the MI+SB203580 group were lower than those in the MI group (Fig. 7D, E). In the AC16 cells treated with SB203580, the level of p38MAPK phosphorylation decreased, autophagy decreased, APOO expression did not change significantly (Fig. 7F-H), and cell viability increased (Fig. 7I), and cytotoxicity decreased (Fig. 7J). The above experimental data suggest that APOO may activate autophagy through the p38MAPK signaling pathway.

## Discussion

MI is a serious problem that has threatened global human health increasingly in recent years. It is the main cause of death and disability worldwide and leads to high medical costs.^16^ The main function of apolipoprotein is to transport plasma lipids to various tissues of the body through the lymphatic and circulatory systems for metabolism and utilization, thus affecting the occurrence and development of diseases such as hyperlipidemia, atherosclerosis, and MI.^17^ In this study, the authors found that the plasma concentration of APOO in patients with MI was significantly higher than that in healthy subjects, and the expression of APOO in the plasma and myocardial tissue of mice with MI was also higher in parallel with the findings in patients. In addition, the expression of APOO in AC16 cells subjected to hypoxia was increased. However, apolipoproteins with different phenotypes have other functions; for instance, APOE activates Akt/PKB phosphorylation.^18^ Moreover, there is structural homology between APOL6 and Bcl-2 family members, and APOL6 can regulate autophagy.^19^ Thus, APOO may be able to participate in the regulation of mitochondrial morphology and function, which may eventually lead to autophagy and apoptosis.^11^^,^^14^

Autophagy is an evolutionarily conserved catabolic process that involves the self-degradation of organelles and cytoplasmic macromolecules^20^ and that plays different roles in mediating cardiomyocyte death.^21^ Autophagy is an important intracellular process that regulates cardiac homeostasis, particularly as a stress response. Autophagy has been shown to play a protective role in the heart's response to ischemia by eliminating damaged mitochondria.^22^ Although generally beneficial, unregulated, and persistent autophagy may be harmful and lead to autophagic death.^23^ Autophagy disorders have been reported to lead to a variety of cardiovascular diseases, including myocardial hypertrophy, MI, and heart failure.^24^ Autophagy can inhibit or enhance apoptosis, depending on the cell type, environment, or stimulation mode.^25^ Studies have shown that when cardiomyocytes are subjected to hypoxia, autophagy occurs earlier, while apoptosis occurs later.^26^ Similarly, Ding et al. observed that autophagy and apoptosis of vascular smooth muscle cells were increased after exposure to 10‒40 μg/mL of oxidized low-density lipoprotein; however, exposure to higher concentrations (≥60 μg/mL) induced high levels of apoptosis, but autophagy decreased.^27^ The present work showed that the expression of Beclin-1, LC3, and Bax proteins in the myocardium of mice with MI increased, the level of Bcl-2 protein decreased, and the concentration of CK-MB and cTnI in the plasma increased. In addition, after the hypoxia treatment of AC16 cells, autophagy was activated, apoptosis increased, cell activity decreased, and cytotoxicity increased in a time-dependent manner. The present results are consistent with those reported by Matsui et al. who found that during cardiac reperfusion, the knockout of Beclin1 is accompanied by a decrease in autophagy and a decrease in cardiac injury.^28^ Excessive autophagy destroys most of the organelles, which further aggravates the pathological death of cardiomyocytes in MI.^29^ However, some studies have shown that upregulation of autophagy may protect cardiomyocytes through AMPK, mTOR, or FOXO signaling pathways, promote mitochondrial clearance, inhibit cell death, slow down heart failure, and reduce myocardial injury.^30^^,^^31^

In addition, to explore the relationship between APOO and autophagy, the authors used APOO knockdown shRNA to block the APOO levels in mice with MI. In the present study, APOO expression was decreased after APOO lentivirus administration; autophagy and apoptosis were reversed. Furthermore, the plasma levels of CK-MB and cTnI improved. In AC16 cells, after silencing the expression of APOO with si-APOO, autophagy and apoptosis were also reduced, cell viability was enhanced, and cytotoxicity was weakened. These results revealed that APOO knockdown reversed autophagy and apoptosis and improved myocardial damage.

The p38MAPK signaling pathway is one of the mitogen-activated protein kinase pathways. In normal immune and inflammatory responses, the p38MAPK signaling pathway is activated to phosphorylate transcription factors and regulate gene expression, participating in a variety of intracellular biological activities.^32^ Some studies have shown that the p38MAPK pathway is activated in MI,^33^^,^^34^ and p38MAPK plays a role in promoting survival and protecting cardiomyocytes during MI.^35^ However, the inhibition of p38MAPK could enhance the protective effect of mesenchymal stem cells in the treatment of MI in animal experiments.^36^ In a double-blind, randomized controlled trial, oral administration of losmapimod, a p38MAPK inhibitor, was found to have potential myocardial protective effects in patients with non-ST segment elevation MI, which might improve the prognosis of the acute coronary syndrome.^37^ p38MAPK is an important factor in balancing apoptosis and autophagy.^38^ Keil et al.^39^ found that p38MAPK may inhibit hunger-induced autophagy through the phosphorylation of Atg5. However, p38MAPK can induce autophagy through Beclin1.^40^ In this study, the levels of p38MAPK phosphorylation were increased in MI-related mouse and cell models but decreased after APOO knockout. In addition, the increase in autophagy caused by MI was reversed after the SB203580 treatment in the mice and AC16 cells.

The main innovation of this study is that APOO may regulate autophagy via the p38MAPK signaling pathway in MI, thus aggravating the myocardial injury. The main limitations of this study are as follows. First, there are few studies on the potential regulatory effect of APOO on autophagy in MI, and more data are needed to confirm the results of this study. Second, there are some deficiencies in the design of this study; for instance, the authors did not explore the effects of APOO overexpression on autophagy. In future research, the authors plan to design experiments to solve the abovementioned problems.

## Conclusions

This study, based on the mouse and AC16 cell models of MI, found that increased APOO levels in mouse myocardial tissue and AC16 cells after MI may activate autophagy and apoptosis by regulating the p38MAPK signaling pathway and, thus, aggravating the myocardial injury. This suggests that the APOO/p38MAPK signaling pathway may be a novel therapeutic approach for MI.

## Authors' contributions

Yue Liu - Conceptualization, data curation, and writing original draft.

Zhiping Xiong - Data curation and formal analysis.

Wei Zhou - Software and validation.

Yuxin Chen - Writing - review & editing.

Qing Huang - methodology and validation.

Yanqing Wu - Supervision, review & editing.

## Funding statements

None.

## Conflicts of interest

The authors declare no conflicts of interest.
